# Initial survey of *PLA2G6* missense variant causing neuroaxonal dystrophy in Papillon dogs in North America and Europe

**DOI:** 10.1186/s40575-020-00098-4

**Published:** 2020-11-30

**Authors:** Karthik Raj, Urs Giger

**Affiliations:** Section of Medical Genetics (PennGen Laboratories), School of Veterinary Medicine, University of Pennsylvania, 3900 Delancey St., Philadelphia, PA 19104-6010 USA

**Keywords:** Canine, Mutations, Screening, Breeding, Ataxia

## Abstract

**Background:**

An autosomal recessive, rapidly progressive degenerative neuropathy known as infantile neuroaxonal dystrophy (NAD) was originally reported in Papillion puppies in 1995. In 2015, a causative missense variant in the *PLA2G6* gene was identified in three affected puppies. Archived samples from Papillons clinically diagnosed with NAD prior to 2015 as well as samples obtained from 660 Papillons from North America and Europe between 2015 and 2017 were screened for the presence of this *PLA2G6* gene variant (XM_022424454.1:c.1579G > A) using a TaqMan assay.

**Results:**

Archived samples from affected puppies diagnosed prior to 2015 and three more recently acquired samples from Papillons clinically affected with NAD were all homozygous for the variant. SIFT analysis predicts that the *PLA2G6* missense substitution (XP_022280162.1:p.Ala527Thr) will not be tolerated in the iPLA_2_β protein. Notably, 17.5% of the 660 tested Papillons were heterozygotes, resulting in a variant allele frequency of 0.092 in this initial survey. Since then, screening for NAD in Papillons by at least 10 other laboratories and data from the Health Committee of Papillon Club of America gathered between 2017 and 2019 reveal a variant allele frequency of 0.047.

**Conclusions:**

This survey and data from other laboratories documents the widespread presence of the *PLA2G6* variant in the Papillon population in North America and Europe. Despite the apparent declining prevalence of the *PLA2G6* variant, screening of Papillons intended for breeding is still recommended to avoid inadvertent production of puppies with infantile NAD.

## Plain English summary

Infantile neuroaxonal dystrophy (NAD) is a rare rapidly progressive disease first reported in Papillion dogs in 1995. Clinical manifestations of ataxia, head tremor, difficulty rising, discordant gate, limb extension, paresis, inability to prehend food and water, and blindness are observed by 1–3 months of age. A specific genetic variant in *PLA2G6* was recently identified in three Papillons with NAD in Japan. We screened Papillons with a clinical diagnosis of NAD received prior to 2015 and samples from an additional 660 Papillons from North America and Europe received between 2015 and 2017 for the presence of this gene variant. All samples from Papillons clinically affected with NAD were homozygous for the variant. Furthermore, 17.5% of all Papillons tested were asymptomatic heterozygotes, and therefore able to pass on the variant to their offspring. Since 2017, an increasing number of laboratories offer NAD screening of Papillons, often together with progressive retinal atrophy testing. Data obtained between 2017 and 19 indicates a variant allele frequency of 0.047. While all these surveys are biased, the apparently high prevalence of the *PLA2G6* variant indicates that screening of Papillons intended for breeding is still recommended to avoid unintentional production of puppies with infantile NAD.

## Background

Hereditary neuropathies in dogs frequently present at an early age and are generally progressive and fatal. Such diseases have been reported in numerous dog breeds (Online Mendelian Inheritance in Animals, OMIA) [1]. Identification of a causative mutation for these genetic diseases allows for a precise diagnosis, and can provide initial insight into therapeutic strategies that may prevent or reduce disease progression. As such, companion animals with hereditary diseases can serve as translational large animal models to investigate the potential safety and efficacy of novel treatments for human disease. However, screening and informed breeding are pivotal to reduce the widespread occurrence of disease-associated allelic variants in future generations of companion dogs.

Neuroaxonal dystrophy (NAD) represents a group of autosomal recessive degenerative neuropathies characterized histopathologically by ‘spheroids’ in the central nervous system and caused by one of a few dysfunctional genes in humans [2] and various animal species including dogs [1, 3]. In humans, variants in the phospholipase A2 group VI (*PLA2G6*) gene, which encodes a calcium-independent enzyme essential for membrane integrity, cause *PLA2G6*-associated neurodegeneration (PLAN) [4, 5].

An infantile NAD (OMIA #: 002105–9615) was first identified in Papillion dogs in 1995 [6–11]. Clinical manifestations of ataxia, head tremor, difficulty rising, discordant gate, limb extension, paresis, inability to prehend food and water, and blindness are observed by 1–3 months of age. Axonal spheroids are found throughout the central nervous system but not in any peripheral nerves, and no iron accumulation was noted [7, 8, 11]. Because clinical signs are rapid and progressive, natural death occurs at a few months of age if not preceded by humane euthanasia. In the absence of a genetic test to identify carriers of this disease, breeders have excluded any Papillon parents and littermates of affected puppies from breeding to reduce the risk of producing affected puppies. Recently, a homozygous missense variant (XM_022424454.1:c.1579G > A) in *PLA2G6* gene was identified in three Papillons with NAD in Japan [11]. In this report, we tested archived samples from Papillons with a clinicopathological diagnosis of NAD for the presence of the *PLA2G6* missense variant and provide results of an initial genotyping survey of Papillons from North America and Europe.

## Materials and methods

The Section of Medical Genetics and PennGen Laboratories had stored samples from four Papillons clinically diagnosed with NAD prior to 2015. These samples, as well as samples from their relatives, were analyzed for the published *PLA2G6* missense variant [11]. Based upon the results, PennGen started to offer a genotyping assay for the *PLA2G6* variant in late 2015. Either cheek swab or EDTA blood samples were accepted for genotyping. Genomic DNA was extracted using QIAamp Blood Mini Kit (Qiagen, Hilden, Germany). A TaqMan genotyping assay, as previously described [11], was used to determine the genotypes.

## Results and discussion

The TaqMan genotyping assay for the XM_022424454.1:c.1579G > A variant in *PLA2G6* readily differentiated the three genotypes. The four Papillons clinically affected with NAD were homozygous for the *PLA2G6* variant, while samples from non-affected dogs were either homozygous for the wild-type allele or heterozygous for the previously published pathogenic variant [11].

Between October 2015 and December 2017, samples from 660 Papillons were received from North America and Europe (Table 1). Only three puppies homozygous for the pathogenic variant were found, with one each from USA, Canada, and Europe. All three Papillon puppies were clinically affected. The low number of affecteds is likely due to the fact that Papillon breeders are well aware of the typical clinical signs of NAD and may not feel the need to submit a sample for testing to confirm their presumptive diagnosis. Among the 660 dogs, 17.5% were heterozygous.

**Table 1:** Genotyping survey results from 2015 to 2017 of Papillon dogs from various geographic regions

Region	Dogs Tested	***PLA2G6*** Genotypes (c.1579G > A)			Variant allele frequency
		Homozygous wild-type, GG	Heterozygous, GA	Homozygous variant, AA	
**United States**	552	461	90	1	0.083
**Canada**	67	51	15	1	0.126
**Europe**	41	29	11	1	0.158
**Total (%)**	660 (100)	541 (81.9)	116 (17.5)	3 (0.4)	0.092

When the genotype screening test became available in late 2015, the demand was large and many breeders were interested in knowing if their Papillon was a carrier. Indeed, 79.5% of all requests indicated breeding and/or general screening as the reason for testing. Following screening, breeders were immediately able to avoid producing affecteds by mating clear (wild type; GG) to clear or clear to carrier (heterozygote; GA). While biased, the ability to undertake more selective breeding appeared to result in a decline in variant allele frequency within the first 3 years of screening (Table 2). As such, screening can help reduce the proportion of carriers in the population, reduce the need for future testing, and permit the breeding of many Papillons, particularly close relatives of an affected puppy that would not have been bred in the absence of testing in order to reduce potential disease risk.

**Table 2:** Change of genotyping results from 2015 to 2017 of Papillon dogs

Year	Dogs Tested	***PLA2G6*** Genotypes (c.1579G > A)			Variant allele frequency
		Homozygous wild-type, GG	Heterozygous, GA	Homozygous variant, AA	
**2015**	97	69	26	2	0.154
**2016**	479	400	78	1	0.083
**2017**	84	72	12	0	0.071
**Total (%)**	660 (100)	541 (81.9)	116 (17.5)	3 (0.4)	0.092

As of late 2017, additional laboratories now offer the test, resulting in a dramatic reduction in the number of samples received by PennGen (only 14 Papillons with 5 carriers over the next 2 years). As of 2020, at least 10 laboratories worldwide (listed by the World Small Animal Veterinary Association, a web resource on DNA tests for hereditary diseases) offer genotyping for this variant as a specific test and/or as part of Papillon breed panel testing or dog panel testing [12, 13]. A summary statement gathered by the Health Committee of Papillon Club of America indicates screening of an additional 2305 Papillons with a variant allele frequency of 0.047 in 2018–2019; this represents a decline of 50% over the prior 3 years. It should be noted that both our survey and that of the Club are biased by at least two factors. First, interested breeders with affected and carrier Papillons preferentially choose to screen their dogs, biasing the survey towards an increased allele frequency. Second, as the signs of juvenile NAD [14] are easily recognizable by breeders and clinicians, affected puppies are likely subject to immediate humane euthanasia. As such puppies may not be tested, the survey could be potentially biased towards a decrease in allele frequency.

Since 1995, there have been a few case reports of NAD in Papillons from the United Kingdom [6] and thereafter from Canada [10] and Japan [7–9, 11] suggesting a widespread distribution of this devastating disease trait in the breed. The Papillon breed has a relatively small breeding pool, with likely common inbreeding and international breedings. As the disease-related gene and variant were only recently identified [11], the prior inability to screen for this genetic disease resulted in its global dissemination. Notably, our results suggest that the widespread availability of screening tests for this *PLA2G6* variant has resulted in successful reduction of the prevalence of the variant allele in the Papilion population.

The point variant XM_022424454.1:c.1579G > A in the *PLA2G6* gene reported to be associated with NAD in Papillons results in an amino acid substitution of the 527th of 806 amino acids from a highly conserved alanine (as far back as zebrafish) to a threonine (XP_022280162.1:p.Ala527Thr) [11]. This missense variant resides in the serine lipase consensus sequence of the patatin domain of the iPLA_2_β protein, and is not tolerated based on SIFT analysis [15]. The overall structure and functional domains of the iPLA_2_β enzyme are highly conserved between dog and human (90.8%) and among other species. As expected for a missense variant, the iPLA_2_β protein was expressed normally in the brain of Papillon puppies with NAD, but was predicted to be severely dysfunctional [11].

Recently, the genotype-phenotype associations in human patients with NAD and *PLA2G6* variants (so called PLAN disorders) were reviewed [4, 5]. Interestingly, missense variants were also commonly reported in human patients with PLAN. However, in humans, variants were found throughout the gene and were not associated with age of disease onset or clinical presentation of the four PLAN subtypes [4, 5]. While the affected Papillons present similarly to children with infantile NAD, an analogous variant has not yet been identified in human patients, although similar missense variants in the same domain are observed [4].

In human medicine, a variant allele frequency of 0.1 (10%) is considered extremely high and is very rarely seen, except in a few select populations for very select variants [16]. While the survey of canine infantile NAD presented here is biased, our findings together with the initial report from Japan, clearly document the worldwide distribution and high prevalence of the variant *PLA2G6* allele in the Papillon population. Such findings are not unusual in specific dog breeds, as common ancestry, founder effects, and international breeding [16] can lead to high frequencies of a genetic disease in afflicted breeds. A common ancestor for the *PLA2G6* gene variant has not been identified.

It should be noted that once a validated pathogenic variant is identified in a population, one can safely breed heterozygotes for the variant (asymptomatic carriers) to homozygous wildtype dogs (clear dogs, normals). Subsequent testing of offspring intended for breeding will then prevent further production of any affected puppies and reduce or eliminate spreading of the variant allele in the breed population. Such strategies also highlight the importance of maintaining a broader gene pool in a small breed population. Although limited in scope, the observed decline between 2015 and 2019 that likely occured as a result of screening of the breeding population and consequent informed breeding practices, shows the keen interest and willingness of breeders to follow practices that will avoid production of any puppies affected by NAD.

In conclusion, this survey from North America and Europe using samples collected between 2015 and 2017, combined with data obtained from other laboratories between 2017 and 2019 documents the widespread presence of the *PLA2G6* variant in the Papillon population. Despite the apparent declining prevalence of the *PLA2G6* variant over the past years, screening of Papillons intended for breeding is recommended unless cleared by parent testing.

## Data Availability

The datasets used and/or analyzed during the current study are available from the corresponding author on reasonable request.

## Abbreviations

NAD: Neuroaxonal dystrophy
PLAN: *PLA2G6*-associated neurodegeneration
OMIA: Online Mendelian Inheritance in Animals
